# ﻿The mitochondrial genome of *Huaaristarchorum* (Heude, 1889) (Gastropoda, Cerithioidea, Semisulcospiridae) and its phylogenetic implications

**DOI:** 10.3897/zookeys.1192.116269

**Published:** 2024-02-22

**Authors:** Yibin Xu, Sheng Zeng, Yuanzheng Meng, Deyuan Yang, Shengchang Yang

**Affiliations:** 1 Key Laboratory of Cultivation and High-value Utilization of Marine Organisms in Fujian Province, Fisheries Research Institute of Fujian, Xiamen, China Key Laboratory of Cultivation and High-value Utilization of Marine Organisms in Fujian Province, Fisheries Research Institute of Fujian Xiamen China; 2 College of the Environment and Ecology, Xiamen University, Xiamen, China Xiamen University Xiamen China; 3 National Taiwan Ocean University, Keelung, Taiwan National Taiwan Ocean University Keelung Taiwan

**Keywords:** *16S* rRNA, *COX1*, mitogenome, phylogenetic analysis, semisulcospirid gastropods

## Abstract

Research on complete mitochondrial genomes can help in understanding the molecular evolution and phylogenetic relationships of various species. In this study, the complete mitogenome of *Huaaristarchorum* was characterized to supplement the limited mitogenomic information on the genus *Hua*. Three distinct assembly methods, GetOrganelle, NovoPlasty and SPAdes, were used to ensure reliable assembly. The 15,691 bp mitogenome contains 37 genes and an AT-rich region. Notably, the cytochrome c oxidase subunit I (*COX1*) gene, commonly used for species identification, appears to be slow-evolving and less variable, which may suggest the inclusion of rapidly evolving genes (NADH dehydrogenase subunit 6 [*ND6*] or NADH dehydrogenase subunit 2 [*ND2*]) as markers in diagnostic, detection, and population genetic studies of Cerithioidea. Moreover, we identified the unreliability of annotations (e.g., the absence of annotations for NADH dehydrogenase subunit 4L [*ND4L*] in NC_037771) and potential misidentifications (NC_023364) in public databases, which indicate that data from public databases should be manually curated in future research. Phylogenetic analyses of Cerithioidea based on different datasets generated identical trees using maximum likelihood and Bayesian inference methods. The results confirm that Semisulcospiridae is closely related to Pleuroceridae. The sequences of Semisulcospiridae clustered into three clades, of which *H.aristarchorum* is one; *H.aristarchorum* is sister to the other two clades. The findings of this study will contribute to a better understanding of the characteristics of the *H.aristarchorum* mitogenome and the phylogenetic relationships of Semisulcospiridae. The inclusion of further mitochondrial genome sequences will improve knowledge of the phylogeny and origin of Cerithioidea.

## ﻿Introduction

The typical animal mitochondrial genome (mt) is a closed-circular molecule ranging from 14 to 20 kilobases (kb) in length and contains 13 protein-coding genes (PCGs), 22 transfer RNA genes (tRNAs), two ribosomal RNA genes (rRNAs, *12S* and *16S*), and a non-coding region (NCR) (Boore 1999). mtDNA is widely used to identify common species and investigate genetic relationships and phylogenetic patterns due to its simple structure, abundant copies, rapid evolutionary rate, and ease of isolation (Mabuchi et al. 2014). However, the absence of complete mitochondrial genome sequences in species belonging to the genus *Hua* creates a gap in molecular biology, potentially resulting in an incomplete understanding of the genus’s phylogenetic relationships and population history.

Semisulcospiridae Morrison, 1952 is a family of freshwater benthic gastropods comprising more than 50 species from four genera (Liu et al. 1993; Du et al. 2019a, 2019b; Lydeard and Cummings 2019). Semisulcospiridae is mainly distributed in East Asia and North America, with most members of this family (43 species from three genera) reported in China (Du et al. 2019a, 2019b). *Hua* S.-F. Chen, 1943 is a genus of freshwater gastropods belonging to Semisulcospiridae, comprising 16 species (Du et al. 2019a, 2019b; Lydeard and Cummings 2019; Strong et al. 2022). This genus is endemic to southwest China and northern Vietnam, and is commonly observed in clean and well-oxygenated water bodies, such as streams, springs, oligotrophic lakes and rivers (Liu et al. 1979). They are commonly used as environmental indicators. Many species of this genus are narrowly distributed; for example, they are found only in certain springs (Du et al. 2019a; Du and Yang 2023). Due to the eutrophication of water bodies, they face the risk of extinction (Strong and Köhler 2009; Du et al. 2019a, 2019b). Moreover, semisulcospirids have been extensively studied for their role as intermediate hosts of some trematodes, such as *Paragonimus* (Davis et al. 1994). *Huaaristarchorum* (Heude, 1888) is a medium-sized species commonly found in the lakes and rivers of southwestern China. As a well-known representative of *Hua* (Du et al. 2019a), mitogenomic data obtained for this species will provide valuable information on the taxonomy of Semisulcospiridae.

Heude (1889) studied freshwater snails of the middle and lower Yangtze River and named 24 species under the genus *Melania* Lamarck, 1799, including *Melaniaaristarchorum* Heude, 1888, the original combination of *Huaaristarchorum*. The genus *Hua* was originally named by Chen (1943), and includes 25 species (five species were named by Heude, as mentioned before), together with the genus *Wanga* S.-F. Chen, 1943, which includes eight species (Chen et al. 2023; Du and Yang 2023). The shells of the genus *Hua* are smooth, whereas those of the genus *Wanga* have sculptures. Chen designated *Melaniatelonaria* Heude, 1888 as the type species of the genus *Hua*, and *Melaniahenriettae* Gray, 1834 as the type species of the genus *Wanga*.

Because so many names have been applied and morphological polymorphisms have been observed in freshwater Cerithoidea (Davis 1972; Minton et al. 2008), the validity of these taxa is doubtful. After the introduction of molecular biology, a portion of this mystery seemed to have been solved. Köhler and Glaubrecht (2001) revised the genus *Brotia* H. Adams, 1866, and proposed the genus *Wanga* as a synonym of the genus *Brotia*, because the type species of *Wanga*, *Melaniahenriettae*, belongs to *Brotia*. Strong and Köhler (2009) raised Semisulcospirinae from a subfamily of Pleuroceridae into an independent family through the morphological and molecular analysis of '*Melaniajacqueti*' Dautzenberg & H. Fischer, 1906, and placed the species into *Hua*. Du et al. (2019a, 2019b) revised the semisulcospirid species in China according to *16S* rRNA and *COX1* genes, and reproductive organs, and demonstrated that there are three genera of Semisulcospiridae in China (*Semisulcospira* O. Boettger, 1886, *Koreoleptoxis* J. B. Burch & Y. Jung, 1988 and *Hua*). In these two studies, *Melaniaaristarchorum* was reclassified as *Hua*.

Previous taxonomic studies on mollusks based on molecular biology have commonly used mitochondrial genes, specifically *COX1*, for species identification, estimation of differentiation rates, and detection of new species (Köhler et al. 2010b; Zhang et al. 2015; Köhler 2017; Aksenova et al. 2018; Du et al. 2019a; Du et al. 2019b; Du and Yang 2019; Wiggering et al. 2019; Yang and Yu 2019; Liang et al. 2022; Wilke et al. 2023; Zhang et al. 2023). Zhang et al. (2018) reported that *COX1* is one of the most conserved PCGs in the mitochondrial genome. Therefore, some species that differ significantly in morphology exhibit only slight differences in their *COX1* gene expression (Köhler et al. 2010a; Du et al. 2019a). Du et al. (2019a) reported that the *p*-distance between *Huaaubryana* (Heude, 1889) and *H.tchangsii* L.-N. Du, Köhler, G.-H. Yu, X.-Y. Chen & J.-X. Yang, 2019 was only 0.9%. Therefore, *COX1* is limited in terms of species identification and phylogenetic studies. To address this problem, complete mitogenome sequencing or the exploration of other mitochondrial PCGs is required.

## ﻿Materials and methods

### ﻿Specimen collection and identification

The studied specimen was collected in the Panlong River, Kunming City, Yunnan Province, China (25°7'14"N, 102°44'50"E). This species is not included on the endangered list of the International Union for Conservation of Nature (https://www.iucnredlist.org/). The specimen was fixed and preserved in 100% ethanol. Tissues were preserved at -20 °C in a refrigerator, and the voucher specimen (No. RTM13) was deposited at the College of the Environment and Ecology, Xiamen University.

A morphological examination and DNA sequence blast confirmed the specimen to be *Huaaristarchorum*. Morphological identification was performed as previously described (Chen 1943; Du et al. 2019a, 2019b). Identifying characteristics were: medium-sized shell, ovate, with four to five whorls; sculpture variable, consisting of four spiral lirae at the base of the shell, three to four spiral lirae on the upper part of the body whorl, and 12 to 13 axial ribs. The mt *COX1* and *16S* rRNA sequences were compared with those in the GenBank database using a BLAST search. Fourteen sequences of *16S* rRNA and 12 sequences of *COX1* exhibited an identity of over 99% (*16S*, GenBank accession No. MK251661, named *H.aristarchorum*) and 99.74% (*COX1*, GenBank accession No. MK251736; *H.aristarchorum*). These 26 sequences corresponded to that of 14 specimens from Huize County and Songming County, Yunnan Province, China (Du et al. 2019b).

### ﻿DNA extraction, mitogenome sequencing and assembly

Muscle tissue (1 mm^3^) was clipped from the foot of the specimen for DNA extraction. A TIANamp Genomic DNA Kit (TIANGEN, Beijing, China) was used to extract whole genomic DNA. The mitogenome of *H.aristarchorum* was sequenced using an Illumina Truseq^TM^ DNA Sample Preparation Kit (Illumina, San Diego, CA, USA) with paired reads measuring 150 bp in length. Quality control of raw genomic data was assessed using FastQC v.0.11.5 (Andrews 2010).

Quality trimming and data filtering were performed using fastp v.0.23.2 (Chen et al. 2018). Trimmed reads containing unpaired reads, more than 5% unknown nucleotides, and more than 50% bases with Q-value ≤ 20 were discarded. To evaluate the consistency of the assembly results, GetOrganelle v.1.7.7.0 (Jin et al. 2020), NovoPlasty v.4.3.1 (Dierckxsens et al. 2017) and SPAdes v.3.15.5 (Bankevich et al. 2012) were used.

### ﻿Mitogenome annotation and sequence analyses

The mitogenome was annotated using the MitoZ annotation module (Meng et al. 2019). The results of the annotation were loaded into Geneious v.2021.0.3 (Kearse et al. 2012) and checked manually with the view of open reading frames (ORFs). Transfer RNA genes were plotted according to the secondary structure predicted by MitoZ v.3.6 (Meng et al. 2019) and MITOS2 (Bernt et al. 2013). The NCR region was determined using the adjacent genes.

**Figure 1.**
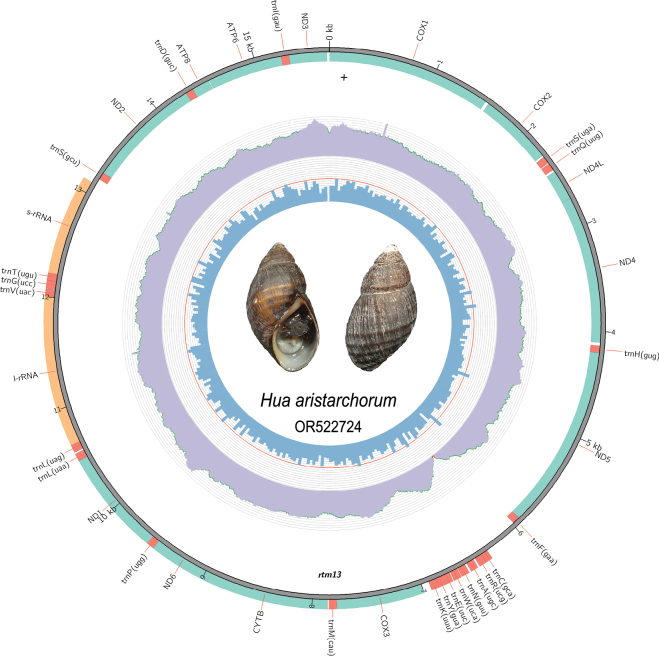
Gene map of the *H.aristarchorum* mitogenome. The photo in the middle is the studied specimen of *H.aristarchorum* (photograph by Yuanzheng Meng). The innermost and middle circles depict the GC content and distribution of the sequencing depth, respectively. The outermost circle represents the arrangement of genes: inner genes from the forward strand, and outer genes from the reverse strand, with the protein-coding genes (PCGs) in green, ribosomal RNAs (rRNAs) in orange, and transfer RNA genes (tRNAs) in red.

The final mitogenome sequence was visualized using the visualization subcommand in MitoZ v.3.6 (Meng et al. 2019), and clean reads were mapped to the gene map (Fig. 1) to show the coverage depth and GC content. Base composition and relative synonymous codon usage (RSCU) were determined using MEGA X (Kumar et al. 2018).

Strand asymmetries were calculated using the following formulae (Perna and Kocher 1995): AT-skew = (A-T) / (A+T); GC-skew = (G-C) / (G+C). DnaSP v.6.0 (Rozas et al. 2017) was used to estimate the nucleotide diversity (Pi) in a sliding window analysis (a sliding window of 100 bp and a step size of 20 bp) and non-synonymous (Ka) / synonymous (Ks) substitution rates of Semisulcospiridae. To investigate the gene order arrangement of the mitogenome sequence, we re-annotated sequences from Semisulcospiridae using our annotation method.

### ﻿Phylogenetic analysis

The newly sequenced mitogenome of *H.aristarchorum* and all available Cerithioidea mitogenomes from GenBank (two sequences without annotation: *Batillariacumingii*MT323103 and *Batillariazonalis*MT363252; one sequence without *ND4L*: *Semisulcospiracoreana*NC_037771) (25 September, 2023) and two outgroup species (Table 1) were used for the phylogenetic analysis using PhyloSuite v.1.2.3 (Zhang et al. 2020). Phylogenetic trees were constructed using three types of datasets: (1) amino acid sequences of the 13 PCGs (AA); (2) all codon positions of the 13 PCGs (PCG123); and (3) the 13 PCGs, excluding the third codon position (PCG12).

**Table 1.** List of 23 species and two outgroups used for phylogenetic analysis.

Species	Family	Length (bp)	A + T (%)	Accession No.	Reference
*Alviniconchaboucheti*	Outgroups	15981	67.7	MT123331	(Lee et al. 2020)
*Epitoniumscalare*	Outgroups	15140	69.4	MK251987	(Guo et al. 2019)
*Batillariazonalis*	Batillariidae	15748	65.3	MT363252	(Yan et al. 2020b)
*Batillariaattramentaria*	Batillariidae	16095	65.3	NC_047187	(Group et al. 2019)
*Batillariacumingii*	Batillariidae	16100	65.6	MT323103	(Yan et al. 2020a)
*Tylomelaniasarasinorum*	Pachychilidae	16632	65.2	NC_030263	(Hilgers et al. 2016)
*Turritellabacillum*	Turritellidae	15868	64.8	NC_029717	(Zeng et al. 2016)
*Maoricolpusroseus*	Turritellidae	15865	63.6	NC_068097	Unpublished
*Pseudocleopatradartevellei*	Paludomidae	15368	63.8	NC_045095	(Stelbrink et al. 2019)
*Tarebiagranifera*	Thiaridae	15555	65.4	MZ662113	(Yin et al. 2022)
*Melanoidestuberculata*	Thiaridae	15821	66.3	MZ321058	(Ling et al. 2022)
*Pirenellapupiformis*	Potamididae	15779	63.2	LC648322	(Kato et al. 2022)
*Cerithideasinensis*	Potamididae	15633	66.8	KY021067	(Xu et al. 2019)
*Cerithideatonkiniana*	Potamididae	15617	63.1	MZ168697	(Yang and Deng 2022)
*Cerithideaobtusa*	Potamididae	15708	63.0	NC_039951	(Nguyen et al. 2018)
*Leptoxisampla*	Pleuroceridae	15591	68.8	KT153076	(Whelan and Strong 2016)
***Huaaristarchorum***	Semisulcospiridae	15691	65.3	OR522724	This study
*Semisulcospiragottschei*	Semisulcospiridae	16101	66.5	MK559478	(Lee et al. 2019)
*Semisulcospiracoreana*	Semisulcospiridae	15398	65.7	NC_037771	(Kim and Lee 2018)
*Koreoleptoxisglobusovalis*	Semisulcospiridae	15866	65.1	LC006055	Unpublished
*Koreoleptoxisnodifila*	Semisulcospiridae	15737	65.8	NC_046494	(Choi et al. 2021)
*Koreoleptoxisnodifila*	Semisulcospiridae	17030	64.4	KJ696780	Unpublished
*Semisulcospiralibertina*	Semisulcospiridae	15432	66.2	NC_023364	(Zeng et al. 2015)
*Koreoleptoxisfriniana*	Semisulcospiridae	15474	66.0	OR567887	Unpublished
*Koreoleptoxisfriniana*	Semisulcospiridae	15544	66.1	OR522723	Unpublished

The extracted PCGs of these sequences were aligned using MAFFT v.7.313 (Katoh and Standley 2013), wherein amino acid sequences were aligned using the normal mode and nucleotide sequences were aligned using the codon model. Gblocks v.0.91 (Castresana 2000) was used to remove ambiguously aligned sequences with default settings (for the length after Gblocks, see Suppl. material 1: table S1).

ModelFinder v.2.2.0 (Kalyaanamoorthy et al. 2017) was used to select the best substitution models of maximum likelihood (ML) and Bayesian inference (BI) analyses. The GTR+F+I+G4 model was selected as the best-fitting model for both ML and BI analyses in the PCG123 and PCG12 datasets; LG+F+I+G4 and mtMAM+F+I+G4 were selected for the AA dataset, under ML and BI respectively.

ML analysis was performed in IQ-TREE v.2.2.2 (Nguyen et al. 2015) under an Edge-linked partition model for 20,000 ultrafast bootstraps. BI analysis was performed using MrBayes v.3.2.7a (Ronquist et al. 2012), with two parallel runs for 2,000,000 generations. Finally, iTOL v.6 (Letunic and Bork 2016) was used to visualize the ML and BI trees.

## ﻿Results and discussion

### ﻿Mitogenome organization

The mitogenome assembly results using GetOrganelle, NovoPlasty and SPAdes were 15,691, 15,675, and 15,694 bp with an average coverage of 125, 59, and 77, respectively. The only difference between the three methods was the length of the NCR. We decided to use the result from GetOrganelle, as this software can generate assembly graphs and is more convenient for other researchers to replicate our assembly results.

The size of the complete mitochondrial genome was 15,691 bp, consisting of 13 PCGs, two rRNAs, 22 tRNAs, and one NCR measuring 346 bp (Fig. 1, Table 2). Nine PCGs (*COX1*, *COX2*, *ND4L*, *ND4*, *ND5*, *ND2*, *ATP8*, *ATP6* and *ND3*), seven tRNAs (*trnS*, *trnQ*, *trnH*, *trnF*, *trnS*, *trnD* and *trnI*), and one NCR are distributed on the heavy (H-) strand, while the other genes are distributed on the light (L-) strand (Table 2, Fig. 1). Overall, the light- and heavy-strand regions within the mitogenome of *H.aristarchorum* were concentrated and characterized by both intergenic (18 intergenic intervals, totaling 384 bp) and overlapping regions (three overlaps, totaling 56 bp) (Table 2). Two typical overlaps occur between PCGs (i.e., 7 bp between *ND4L* and *ND4*, and 47 bp between *CYTB* and *ND6*), and these overlaps are common in other freshwater gastropod sequences (Lee et al. 2019). Similar to the mitochondrial genes in other Cerithioidea species (Zeng et al. 2015; Kim and Lee 2018; Choi et al. 2021), the mitochondrial genes in *H.aristarchorum* exhibit a high A + T content of 65.3% (Table 1), with A, T, G, and C constituting 30.8%, 34.5%, 17.9%, and 16.8%, respectively (Table 3). Both the AT- and GC-skew of the mitogenome are negative, -0.056 and -0.032, respectively (Table 3), indicating that Ts and Cs are more abundant than As and Gs.

**Table 2.** Features of the *H.aristarchorum* mitogenome.

Gene	Position		Length (bp)	Amino	Start/stop codon	Anticodon	Intergenic region	Strand
	From	To						
*COX1*	1	1533	1533	511	ATG/TAA		32	H
*COX2*	1566	2255	690	230	ATG/TAA		19	H
*trnS* (uga)	2275	2341	67			TGA	10	H
*trnQ* (uug)	2352	2419	68			TTG	24	H
*ND4L*	2444	2734	291	97	ATG/TAA		-7	H
*ND4*	2728	4095	1368	456	GTG/TAA		35	H
*trnH* (gug)	4131	4196	66			GTG	0	H
*ND5*	4197	5915	1719	573	ATG/TAA		2	H
*trnF* (gaa)	5918	5985	68			GAA	0	H
NCR	5986	6331	346				0	H
*trnC* (gca)	6332	6393	62			GCA	1	L
*trnR* (ucg)	6395	6461	67			TCG	19	L
*trnA* (ugc)	6481	6548	68			TGC	20	L
*trnN* (guu)	6569	6641	73			GTT	7	L
*trnW* (uca)	6649	6717	69			TCA	9	L
*trnE* (uuc)	6727	6791	65			TTC	3	L
*trnY* (gua)	6795	6861	67			GTA	0	L
*trnK* (uuu)	6862	6930	69			TTT	69	L
*COX3*	7000	7779	780	260	ATG/TAA		3	L
*trnM* (cau)	7783	7852	70			CAT	8	L
*CYTB*	7861	9000	1140	380	ATG/TAG		-47	L
*ND6*	8954	9505	552	184	ATG/TAA		2	L
*trnP* (ugg)	9508	9573	66			TGG	4	L
*ND1*	9578	10516	939	313	ATG/TAA		0	L
*trnL* (uaa)	10517	10583	67			TAA	16	L
*trnL* (uag)	10600	10669	70			TAG	0	L
*l-rRNA* (*16S*)	10670	12007	1338				0	L
*trnV* (uac)	12008	12076	69			TAC	5	L
*trnG* (ucc)	12082	12150	69			TCC	1	L
*trnT* (ugu)	12152	12218	67			TGT	-2	L
*s-rRNA* (*12S*)	12217	13107	891				61	L
*trnS* (gcu)	13169	13236	68			GCT	0	H
*ND2*	13237	14304	1068	356	ATG/TAA		0	H
*trnD* (guc)	14305	14373	69			GTC	3	H
*ATP8*	14377	14538	162	54	ATG/TAG		9	H
*ATP6*	14548	15243	696	232	ATG/TAA		2	H
*trnI* (gau)	15246	15316	71			GAT	1	H
*ND3*	15318	15671	354	118	ATG/TAG		19	H

**Table 3.** Composition and skewness of the *H.aristarchorum* mitogenome.

	A%	T%	G%	C%	(A + T)%	AT-skew	GC-skew	Length (bp)
Mitogenome	30.8	34.5	17.9	16.8	65.3	-0.056	-0.032	15691
PCGs	26.2	38.6	18.5	16.7	64.8	-0.192	-0.051	11292
*COX1*	25.7	37.4	18.8	18.1	63.1	-0.185	-0.018	1533
*COX2*	28.4	35.9	18	17.7	64.3	-0.117	-0.008	690
*ND4L*	26.1	39.5	15.8	18.6	65.6	-0.204	0.08	291
*ND4*	27.5	38.2	19.9	14.4	65.7	-0.164	-0.16	1368
*ND5*	28.1	37.8	19.8	14.4	65.9	-0.147	-0.158	1719
*COX3*	24.2	36.2	19.7	19.9	60.4	-0.197	0.003	780
*CYTB*	24.8	37.6	21	16.6	62.4	-0.205	-0.117	1140
*ND6*	27.9	39.1	17	15.9	67	-0.168	-0.033	552
*ND1*	25	39.4	17.7	17.9	64.4	-0.223	0.006	939
*ND2*	25.2	41.7	15.3	17.9	66.9	-0.246	0.079	1068
*ATP8*	31.5	40.7	13.6	14.2	72.2	-0.128	0.022	162
*ATP6*	21.8	43.1	18.4	16.7	64.9	-0.327	-0.049	696
*ND3*	26.8	40.7	15.3	17.2	67.5	-0.205	0.061	354
*l-rRNA* (*16S*)	35.4	31.8	15.3	17.6	67.2	0.053	0.068	1338
*s-rRNA* (*12S*)	32.9	31.9	15.9	19.3	64.8	0.016	0.096	891
tRNAs	32.6	31.6	16.3	19.5	64.2	0.015	0.088	1495
NCR	39.0	24.9	18.2	17.9	63.9	0.222	-0.008	346

### ﻿Genes and codon usage

The mitogenome of *H.aristarchorum* displays the standard arrangement of 13 PCGs commonly observed in Cerithioidea species. These include seven NADH dehydrogenases (*ND1*-*ND6* and *ND4L*), three cytochrome c oxidases (*COX1*-*COX3*), two ATPases (*ATP6* and *ATP8*) and one cytochrome b (*CYTB*). These 13 PCGs have a total length of 11,292 bp and encode 3,764 amino acids. With the exception of *ND4*, which starts with the GTG codon, all others begin with ATG. As for the stop codons, *CYTB*, *ATP8* and *ND3* end with the TAG codon, and the others end with TAA (Table 2), whereas in the sequences of the 13 PCGs within the same family, most genes start with the codon ATG and end with the codon TAA. The AT- and GC-skews of the 13 PCGs are similarly negative, -0.192 and -0.051, respectively (Table 3). Five PCGs (*ND1*, *ND2*, *ND4L*, *COX3* and *ATP8*) exhibit positive GC-skew values, whereas the remaining eight PCGs exhibit negative values.

The *12S* rRNA (891 bp) gene is located between the trnT and trnS genes, and the *16S* rRNA (1,338 bp) gene is located between *trnL* and *trnV* (Table 2, Fig. 1). A total of 22 tRNA genes with lengths ranging from 62 to 73 bp were identified in the mitogenome of *H.aristarchorum*. Most of these tRNA genes exhibit a characteristic cloverleaf-like structure, except for *trnS*, which lacks a dihydrouridine arm (Suppl. material 1: fig. S1).

**Figure 2.**
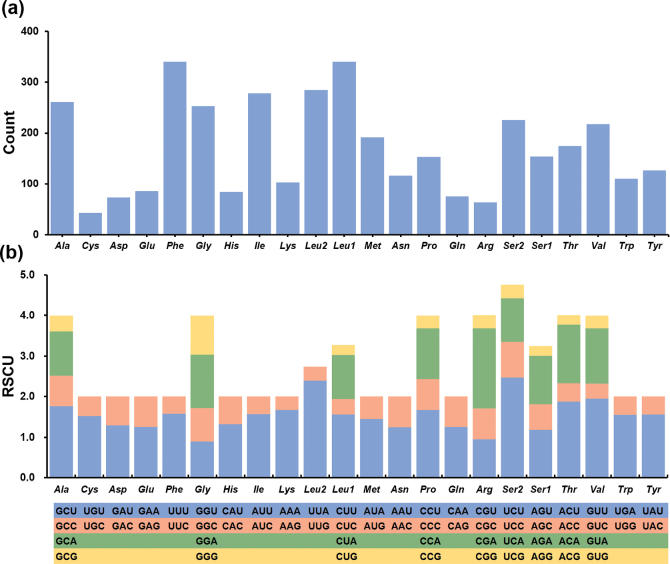
Amino acid composition (**a**) and relative synonymous codon usage (**b**) of the *H.aristarchorum* mitogenome. The codon families are provided under the x-axis.

The relative synonymous codon usage (RSCU) values of the mitogenome were calculated and are summarized in Suppl. material 1: table S2, Fig. 2. Among the 13 PCGs, the most frequently found amino acids are *Leu* (15.57%), *Ser* (10.31%), *Phe* (9.57%) and *Ile* (8.11%). The least common amino acids are *Cys* (1.06%), *Arg* (1.63%), *Gln* (1.76%) and *Asp* (1.90%) (Fig. 2a, Suppl. material 1: table S2). RSCU analysis reveals that the most frequently found codons include UCU (*Ser*), UUA (*Leu*) and CGA (*Arg*), whereas CUG (*Leu*), ACG (*Thr*) and AGG (*Ser*) have the lowest frequencies (Fig. 2b, Suppl. material 1: table S2). RSCU analysis also indicated that codons are biased toward more A/U at the third codon, which is consistent with other Cerithioidea species (Lee et al. 2019; Choi et al. 2021).

### ﻿Nucleotide diversity and evolutionary rate analysis

**Figure 3.**
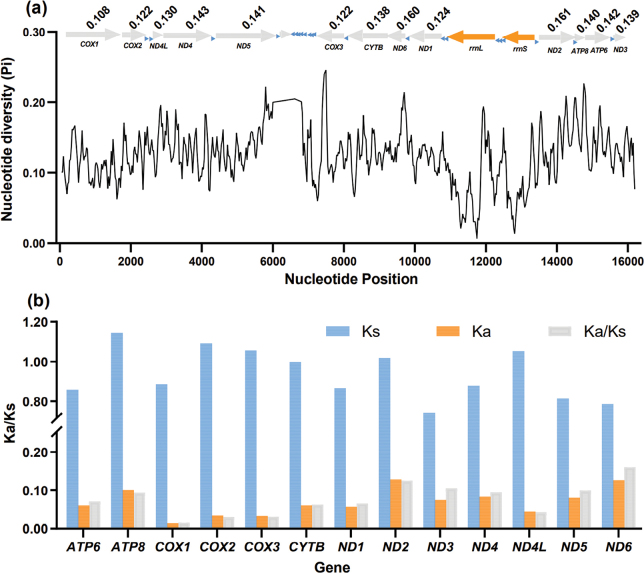
Nucleotide diversity analysis (**a**) and Ka/Ks rates (**b**) of 13 PCGs based on nine Semisulcospiridae species. The Pi values for the 13 PCGs is shown in the graph, with the PCGs in gray, rRNAs in orange, and tRNAs in blue. The black line represents the value of nucleotide diversity (Pi) (window size = 100 bp, step size = 20 bp). The blue, orange and gray columns represent the Ks, Ka and Ka/Ks values, respectively.

Nucleotide diversity analysis (Pi values) among the 13 aligned PCGs in the semisulcospirid mitogenomes revealed a substantial degree of variation within various genes (Fig. 3a). Pi values ranged from 0.108 (*COX1*) to 0.161 (*ND2*). Among all PCGs, *ND2* (Pi = 0.161) exhibited the highest variability, followed closely by *ND6* (Pi = 0.160) and *ND4* (Pi = 0.143). Conversely, *COX1* (Pi = 0.108), *COX2* (Pi = 0.122) and *COX3* (Pi = 0.122) displayed relatively low nucleotide diversity, indicating conservation among the 13 PCGs (Fig. 3a). These observations are also reflected in the Ka/Ks ratios (Fig. 3b). These results indicate that the 13 PCGs from all Semisulcospiridae mitogenomes evolved under purifying selection (Fig. 3). Among these 13 PCGs, *COX1* (Ka/Ks = 0.015) underwent the strongest purifying selection and exhibited the lowest evolutionary rate. In contrast, *ND6* (Ka/Ks = 0.160) and *ND2* (Ka/Ks = 0.125) experienced comparatively weak purifying pressures, indicating a relatively rapid evolutionary rate.

### ﻿Comparative analysis of mitochondrial genome components in Semisulcospiridae

**Figure 4.**
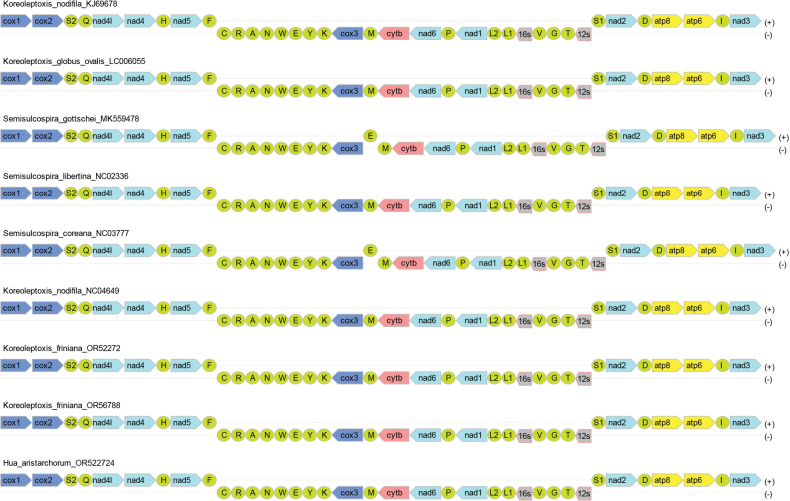
The mitochondrial genome composition and arrangement of Semisulcospiridae. The PCGs are colored based on their functional group (dark blue represents *COX1-3*, light blue corresponds to *ND1-6*, pink indicates *CYTB* and yellow signifies *ATP6* and *ATP8*), rRNAs (*12S* and *16S*) are represented by gray modules, and the positions of the tRNAs are portrayed using their single-letter amino acid code (green modules). The non-coding region is not displayed. Note: *H.aristarchorum* is highlighted in red.

We compared the mitochondrial genome of *H.aristarchorum* with those of other Semisulcospiridae species. After analyzing sequences downloaded directly from GenBank, we found that the gene positions were mostly identical. However, *S.coreana*NC_037771 did not contain *ND4L*, and there were variations in the orientation of certain genes (Fig. 4a). Notably, *K.globusovalis*LC006055, *S.libertina*NC_023364 and *K.nodifila*NC_046494 exhibited different gene orientations, specifically *trnL* in *K.globusovalis*LC006055 and *S.libertina*NC_023364, and *rrnL* in *K.nodifila*NC_046494, which were located on the positive strand (Fig. 4a). After the re-annotation of all sequences within this family, both gene positions and orientations were found to be consistent, indicating a highly conserved gene arrangement (Fig. 4b).

In Semisulcospiridae species, duplicated *trnL* was positioned immediately after *rrnL*, and *trnS* preceded *rrnS* and *ND2*. Additionally, *trnI*, *trnP* and *trnH* were located immediately before *ND3*, *ND6* and *ND5*. Furthermore, *trnM* was located immediately after the *COX3* gene (Fig. 4b). The mitochondrial genome exhibited a highly conserved gene arrangement. These orders were *COX1*-*COX2*, *ND4L*-*ND4*-*ND5*, *ND1*-*ND6*-*CYTB*-*COX3* and *ND2*-*ATP8*-*ATP6*-*ND3*. The type of tRNA between certain PCGs was a common feature in all species of Semisulcospiridae (Fig. 4b).

### ﻿Phylogenetic analysis

**Figure 5.**
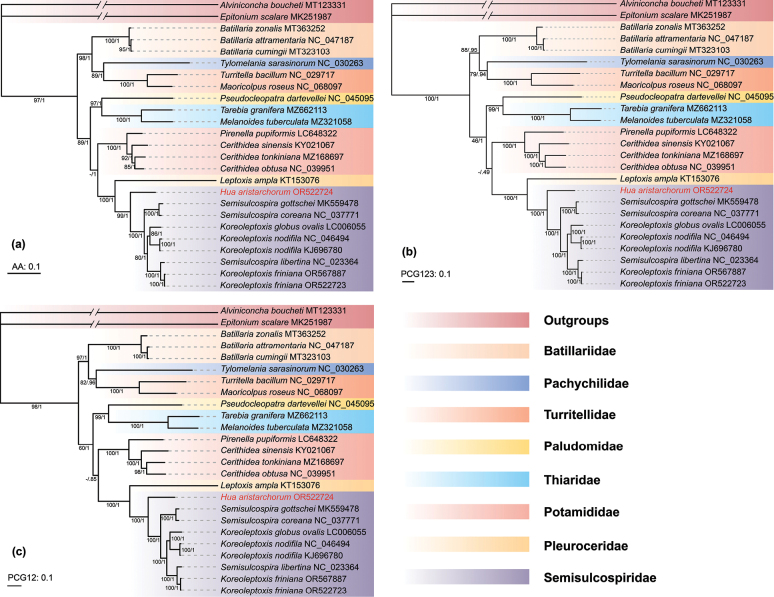
Phylogenetic tree (BI) of Cerithioidea species inferred from dataset I AA (**a**), II PCG123 (**b**) and III PCG12 (**c**). The numbers at the internodes represent maximum likelihood (ML) bootstrap (BS) and Bayesian inference (BI) posterior probabilities (PP). The GenBank accession numbers used are listed after the species names. The scale bar indicates the number of substitutions per site. Note: *H.aristarchorum* is highlighted in red.

In this study, both the ML and BI methods produced identical topological structures for each dataset. The BI tree is presented here due to its higher overall support values. Datasets I (AA), II (PCG123) and III (PCG12) formed a consistent tree (Fig. 5). Among the six trees, the Bayesian posterior probability of the phylogenetic tree based on the AA dataset was the highest (Fig. 5a).

Eight of the 22 extant families belonging to Cerithioidea were included in this study. All Semisulcospiridae species clustered into a clade. These results confirm that Semisulcospiridae is a sister group of Pleuroceridae (Fig. 5a). Within Semisulcospiridae, three of the four genera were included. The nine sequences cluster into three clades, each exhibiting high support. *Huaaristarchorum* is sister to two clades containing *Koreoleptoxis* species; however, a *Semisulcospira* species (*S.libertina* NC023 364) appears among the *Koreoleptoxis* species (Fig. 5a).

We assumed that *S.libertina*NC_023364 may have been misidentified. The distribution of freshwater snails is usually restricted (Von Rintelen and Glaubrecht 2005; Köhler 2017). The type localities of *S.libertina* are Simoda and Ousima in Japan, but specimen KF736848 originated from Poyang Lake, China (Gould 1859; Zeng et al. 2015). The mt *COX1* and *16S* rRNA sequences of NC_023364 were compared with those in the public database GenBank using a BLAST search to verify its exact affiliation. Three sequences of *16S* rRNA and two sequences of *COX1* were matched, exhibiting an identity of over 99%. The identity of the three matching *16S* rRNA sequences was 99.09% (GenBank accession No’s. MK944155, MK944156, and MK944157, from *K.praenotata*), and the two matching *COX1* sequences were 99.21% and 99.08% (GenBank accession No. MK968983, from *K.praenotata*, and MK969039, from *K.davidi*). The specimens were obtained from Wuyuan County, Jiangxi Province (MK944155, MK944156, MK944157, MK969039) and Ningguo City, Anhui Province (MK969893), China (Du et al. 2019b). Based on these findings, we suspect that NC_023364 as *S.libertina* is a misidentification; it should be *K.praenotata* or *K.davidi*.

Many previous studies (Köhler 2017; Du et al. 2019a, 2019b; Du and Yang 2023) indicated that three valid genera are distributed in Asia, as indicated by the three clades shown in the phylogenetic tree (Fig. 5). However, Köhler (2017) considered that *Semisulcospira* is not a monophyletic group; of the three primary clades, two of them are viviparous, and one is oviparous. The oviparous clade is treated as a distinct genus, *Koreoleptoxis*. The other two clades are both classified as *Semisulcospira*, although they do not form a monophyletic group. However, the species involved in this study only cover two of the clades in Köhler (2017). According to these sequences, species relationships are not contrary to Köhler (2017); therefore, due to taxon sampling limitations the conclusion in Köhler (2017) has not been refuted. More sequences and further analysis are still needed to resolve relationships within *Semisulcospira*.

## ﻿Conclusions

In this study, we determined and described the complete mitogenome of *Huaaristarchorum* to supplement the limited mitogenome information available for the genus. Three distinct assembly methods were employed to ensure reliability of the assembly: GetOrganelle, NovoPlasty and SPAdes. The 15,691 bp mitogenome contains 37 genes and an AT-rich region. *ND4* starts with GTG, and the other PCGs start with ATG. All of the PCGs are terminated using the TAN codon. RSCU analysis indicated that codons are biased toward the use of A/U at the third codon.

Nucleotide diversity analysis can help identify regions with significant nucleotide differences, which is useful for species-specific marker development, especially in challenging-to-identify taxa. Our results reveal that the *COX1* gene is the slowest evolving and least variable region, indicating that *COX1* as a barcode may need to be carefully tested. To identify the intricate shell sculpture of species of Semisulcospiridae or other families of Cerithioidea, we suggest the inclusion of genes with rapidly evolving rates and high Pi values, such as *ND6* or *ND2*, may be markers in diagnostic, detection, and population genetic studies of Cerithioidea.

Lee et al. (2019) mentioned concerns regarding the reliability of sequence annotation information in their study of the gene structure of Cerithioidea. This underscores the significance of mitochondrial gene annotation and the need for a uniform annotation approach. In this study, we uniformly annotated all sequences from Semisulcospiridae. In contrast to the partial gene variations that information downloaded directly from GenBank may show, our results revealed a very high level of conservation in gene structure within this family.

ML and BI methods were employed to evaluate phylogenetic relationships within Cerithioidea based on three datasets (AA, PCG123, and PCG12), yielding identical trees. The results confirm that Semisulcospiridae is closely related to Pleuroceridae, and high supports indicate that nine sequences of seven species from three genera used in this study within Semisulcospiridae form three clades, corresponding to three valid genera distributed in Asia. One clade is *H.aristarchorum*, and it is sister to the other two clades. But we find one species (*S.libertina*NC_023364) misplaced. Through analysis of its geographical distribution and comparisons with GenBank database sequences, we suspect that NC_023364 has been misidentified.

Köhler (2017) mentioned that *Semisulcospira* might not be a monophyletic group, but considering the present study only includes nine sequences from seven species, we can only reach a tentative conclusion on genus monophyly. Sequences from more species are still needed to understand the phylogeny of Semisulcospiridae in depth.

In this study, we identified annotation errors and misidentifications in public databases and highlighted their potential influence on our research results. For future research, it is crucial to adopt an appropriate approach that utilizes data from public databases. Moreover, inaccurate phylogenetic inferences are more likely to occur without sufficient specimen acquisition for intraspecific variability and geographic coverage. Therefore, comprehensive taxon sampling is necessary to resolve the phylogeny and origin of Cerithioidea with high accuracy.

## ﻿Additional information

### Author ORCIDs

Yibin Xu https://orcid.org/0009-0004-7386-806X

Sheng Zeng https://orcid.org/0009-0001-0943-3772

Yuanzheng Meng https://orcid.org/0009-0006-3294-8973

Deyuan Yang https://orcid.org/0000-0003-3735-9909

Shengchang Yang https://orcid.org/0000-0003-3731-7872

### Data availability

All of the data that support the findings of this study are available in the main text or Supplementary Information.

## Supplementary materials

Supplementary material 1 Supplementary information Data type docx Explanation note **table S1.** Original and Gblock lengths of the PCG and AA sequences. **table S2.** Codon numbers and relative synonymous codon usage (RSCU) of 13 PCGs in the *H.aristarchorum* mitogenome. **figure S1.** Potential secondary structures of 22 inferred tRNAs in the *H.aristarchorum* mitogenome. https://binary.pensoft.net/file/990893This dataset is made available under the Open Database License (http://opendatacommons.org/licenses/odbl/1.0/). The Open Database License (ODbL) is a license agreement intended to allow users to freely share, modify, and use this Dataset while maintaining this same freedom for others, provided that the original source and author(s) are credited.Yibin Xu, Sheng Zeng, Yuanzheng Meng, Deyuan Yang, Shengchang Yang
