# Supplementary material for: ﻿The mitochondrial genome of Huaaristarchorum (Heude, 1889) (Gastropoda, Cerithioidea, Semisulcospiridae) and its phylogenetic implications
Source: Zookeys. 2024 Feb 22;1192:237–55. doi: 10.3897/zookeys.1192.116269 (PMC10905624; doi:10.3897/zookeys.1192.116269)
Supplement: Supplementary material 1 — Supplementary information [file zookeys-1192-237_article-116269__-s001.docx]

Supplementary Materials for

**The first mitochondrial genome of *Hua aristarchorum* (Heude, 1889) (Gastropoda: Cerithioidea: Semisulcospiridae) and its phylogenetic implications**

Yibin Xu^1*^, Sheng Zeng^2^, Yuanzheng Meng^2^, Deyuan Yang^2,3^, and Shengchang Yang^2*^

Sheng Zeng and Yibin Xu contributed equally to this manuscript

Corresponding authors: Yibin Xu ([3208871@qq.com](mailto:3208871@qq.com)); Shengchang Yang ([scyang@xmu.edu.cn](mailto:scyang@xmu.edu.cn))

**1** Key Laboratory of Cultivation and High – value Utilization of Marine Organisms in Fujian Province, Fisheries Research Institute of Fujian, Xiamen, China

**2** College of the Environment and Ecology, Xiamen University, Xiamen, China **3** National Taiwan Ocean University, Keelung, Taiwan, China.

**This file includes:**

Table S1 – S2, Figure S1

**Table S1.** Original and Gblock lengths of the PCG and AA sequences

| Gene | Original length | | Gblocks length | | Percentage (%) | |
| --- | --- | --- | --- | --- | --- | --- |
|  | PCG | AA | PCG | AA | PCG | AA |
| *ATP6* | 705 | 235 | 693 | 231 | 98.3 | 98.3 |
| *ATP8* | 177 | 59 | 150 | 37 | 84.7 | 62.7 |
| *COX1* | 1533 | 511 | 1529 | 510 | 99.7 | 99.8 |
| *COX2* | 687 | 229 | 686 | 227 | 99.9 | 99.1 |
| *COX3* | 780 | 259 | 771 | 259 | 98.8 | 100.0 |
| *CYTB* | 1137 | 379 | 1137 | 379 | 100.0 | 100.0 |
| *ND1* | 942 | 314 | 931 | 310 | 98.8 | 98.7 |
| *ND2* | 1077 | 359 | 1064 | 337 | 98.8 | 93.9 |
| *ND3* | 351 | 117 | 351 | 117 | 100.0 | 100.0 |
| *ND4* | 1380 | 460 | 1361 | 448 | 98.6 | 97.4 |
| *ND4L* | 321 | 107 | 276 | 91 | 86.0 | 85.0 |
| *ND5* | 1719 | 573 | 1712 | 565 | 99.6 | 98.6 |
| *ND6* | 549 | 183 | 547 | 181 | 99.6 | 98.9 |

**Table S2.** Codon numbers and relative synonymous codon usage (RSCU) of 13 PCGs in the *H. aristarchorum* mitogenome

| Codon | Count | RSCU | Codon | Count | RSCU | Codon | Count | RSCU | Codon | Count | RSCU |
| --- | --- | --- | --- | --- | --- | --- | --- | --- | --- | --- | --- |
| UUU(F) | 268 | 1.58 | UCU(S) | 117 | 2.47 | UAU(Y) | 98 | 1.56 | UGU(C) | 33 | 1.53 |
| UUC(F) | 72 | 0.42 | UCC(S) | 41 | 0.87 | UAC(Y) | 28 | 0.44 | UGC(C) | 10 | 0.47 |
| UUA(L) | 249 | 2.39 | UCA(S) | 51 | 1.08 | UAA(*) | 10 | 1.54 | UGA(W) | 85 | 1.55 |
| UUG(L) | 36 | 0.35 | UCG(S) | 16 | 0.34 | UAG(*) | 3 | 0.46 | UGG(W) | 25 | 0.45 |
| CUU(L) | 162 | 1.56 | CCU(P) | 64 | 1.67 | CAU(H) | 56 | 1.33 | CGU(R) | 15 | 0.95 |
| CUC(L) | 40 | 0.38 | CCC(P) | 29 | 0.76 | CAC(H) | 28 | 0.67 | CGC(R) | 12 | 0.76 |
| CUA(L) | 112 | 1.08 | CCA(P) | 48 | 1.25 | CAA(Q) | 47 | 1.25 | CGA(R) | 31 | 1.97 |
| CUG(L) | 26 | 0.25 | CCG(P) | 12 | 0.31 | CAG(Q) | 28 | 0.75 | CGG(R) | 5 | 0.32 |
| AUU(I) | 218 | 1.57 | ACU(T) | 82 | 1.87 | AAU(N) | 72 | 1.24 | AGU(S) | 56 | 1.18 |
| AUC(I) | 60 | 0.43 | ACC(T) | 20 | 0.46 | AAC(N) | 44 | 0.76 | AGC(S) | 30 | 0.63 |
| AUA(M) | 139 | 1.45 | ACA(T) | 63 | 1.44 | AAA(K) | 86 | 1.67 | AGA(S) | 57 | 1.2 |
| AUG(M) | 53 | 0.55 | ACG(T) | 10 | 0.23 | AAG(K) | 17 | 0.33 | AGG(S) | 11 | 0.23 |
| GUU(V) | 106 | 1.95 | GCU(A) | 115 | 1.76 | GAU(D) | 47 | 1.29 | GGU(G) | 57 | 0.9 |
| GUC(V) | 20 | 0.37 | GCC(A) | 49 | 0.75 | GAC(D) | 26 | 0.71 | GGC(G) | 52 | 0.82 |
| GUA(V) | 74 | 1.36 | GCA(A) | 72 | 1.1 | GAA(E) | 53 | 1.25 | GGA(G) | 83 | 1.31 |
| GUG(V) | 17 | 0.31 | GCG(A) | 25 | 0.38 | GAG(E) | 32 | 0.75 | GGG(G) | 61 | 0.96 |


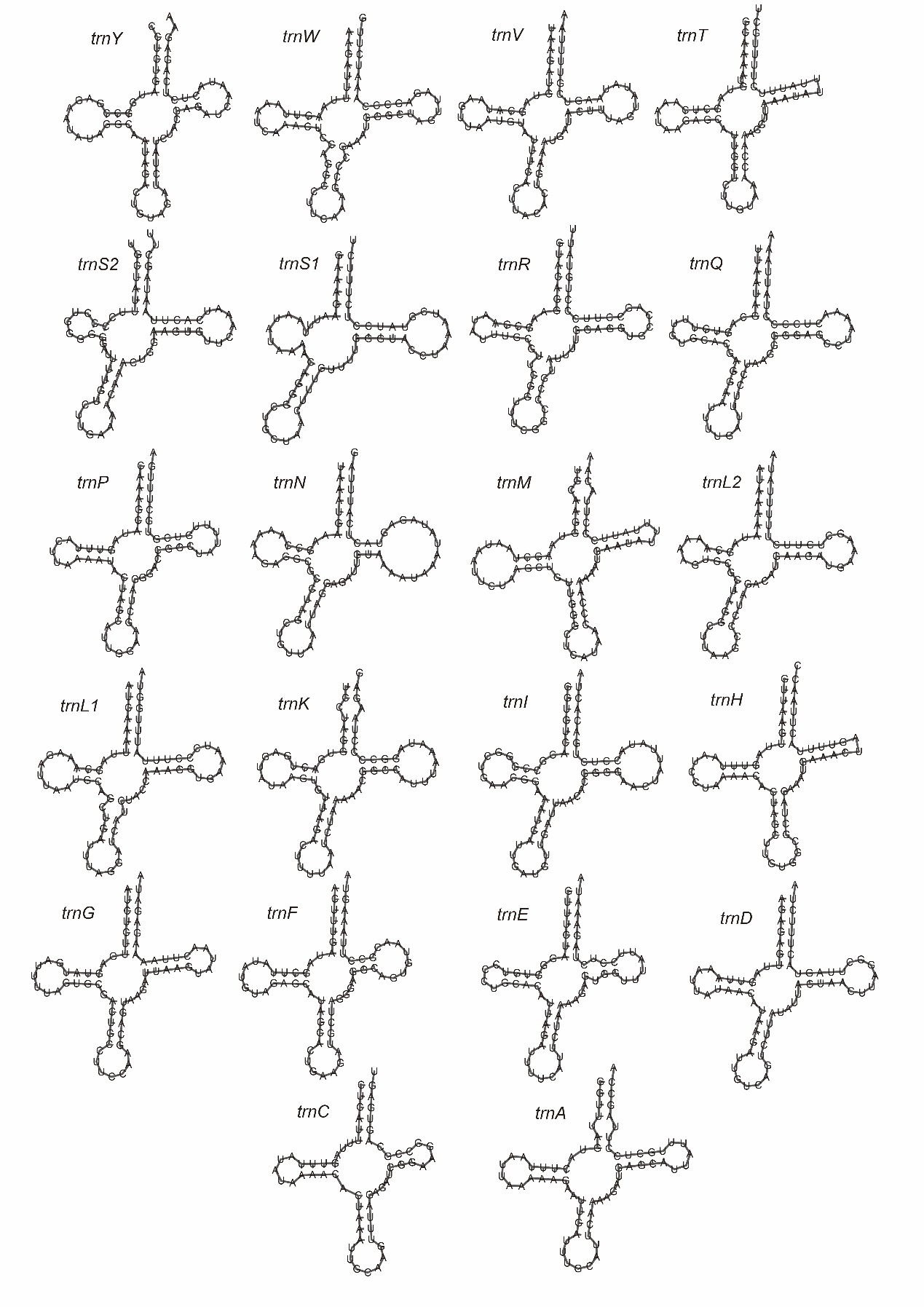


**Figure S1.** Potential secondary structures of 22 inferred tRNAs in the *H. aristarchorum* mitogenome
